# Pragmatic questionnaire-based evaluation of auditory function in individuals with major neurocognitive disorders and hearing loss in diverse contexts

**DOI:** 10.3389/fnagi.2025.1504358

**Published:** 2025-06-13

**Authors:** Panagiotis Alexopoulos, Antonios Alexandros Demertzis, Panagiotis Biris, Polychronis Economou, Eric Frison, Piers Dawes, Iracema Leroi

**Affiliations:** ^1^Faculty of Medicine, Mental Health Services, School of Health Sciences, University of Patras, Patras, Greece; ^2^Global Brain Health Institute, School of Medicine, Trinity College Dublin, Dublin, Ireland; ^3^Department of Psychiatry and Psychotherapy, Klinikum rechts der Isar, Technical University of Munich, Munich, Germany; ^4^Patras Dementia Day Care Centre, Patras, Greece; ^5^Faculty of Medicine, School of Health Sciences, University of Patras, Patras, Greece; ^6^Department of Civil Engineering (Statistics), School of Engineering, University of Patras, Patras, Greece; ^7^Univ. Bordeaux, INSERM, Institut Bergonié, CHU Bordeaux, Bordeaux, France; ^8^University of Queensland Centre for Hearing Research (CHEAR), School of Health and Rehabilitation Sciences, The University of Queensland, Brisbane, QLD, Australia

**Keywords:** dementia, hearing loss, auditory assessment, low-resource settings, questionnaires

## Abstract

**Background:**

Hearing impairment in older people is a significant risk factor for cognitive decline and dementia, while it is a source of bias in the diagnostic workup of cognitive complaints. Early detection and intervention are critical, yet audiometric equipment is often unavailable in primary healthcare- and/or community care-, as well as in low-resource settings across the globe.

**Objective:**

This study aims (i) to develop brief accurate instruments for capturing hearing loss severity based on items of the 25-item Hearing Handicap Inventory for the Elderly (HHIE) and its counterpart the Hearing Handicap Inventory for the communication partner (HHIE-SP) and (ii) to compare their usefulness as well as that of the 10-item screening version of HHIE (HHIE-S) in detecting hearing loss severity in people with dementia and hearing loss to HHIE and HHIE-SP.

**Methods:**

The study relies on screening- and baseline data of the Sense-Cog Trial, being a European, multi-center, observer-blind, 36-week long, randomized controlled trial (RCT) of people with dementia with sensory impairment and their companions. An exploratory data analysis was utilized to provide a comprehensive understanding of the data structure and the characteristics of the sample. Eight different proportional odds logistic regression models were computed to study the relationship between the pure-tone audiometry screen results and different versions of the HHIE, with or without consideration of demographic data of the person with dementia and his/her communication partner. Stratified repeated random subsampling was employed to create two new HHIE models. All models were assessed by calculating the Mean Squared Deviation (MSE) over 1,000 splits into 90% training and 10% test set.

**Results:**

Two separate HHIE-mini models were developed. HHIE-2 includes one item of the HHIE and one item of the HHIE-SP. HHIE-8 includes three items of the HHIE and five items of the HHIE-SP. The model including HHIE-S and demographic data demonstrated the highest performance (MSE = 6.818), followed by the model including HHIE-SP and demographic data (MSE = 7.065) and the HHIE-2 model which included age (MSE = 7.254) but not country of residence. The HHIE-8 model was less effective (MSE = 7.740), and the model including HHIE and no demographic data was the least reliable (MSE = 9.220).

**Conclusion:**

HHIE-S and HHIE-2 combined with demographic data are practical and more efficient tools for assessing hearing loss severity in people with dementia and hearing impairment compared to HHIE, HHIE-S and HHIE-SP in different European countries. They both address the specific challenges associated with dementia-related hearing assessments by limiting the cognitive load of the evaluation process. Particularly the ultra-brief HHIE-2 may be feasible for use in primary and community healthcare settings in different countries, since in a European cohort it is not affected by the country of residence of the individuals with dementia.

## Introduction

The impact of age-related sensory loss on dementia risk, quality of life of patients with dementia and their care partners’ burden is attracting increasing scientific attention. Dementia and hearing loss belong to the 30 leading causes of years lived with disability at a global level, posing an enormous burden to health care systems, families, and societies (Vos et al., 2012). Interestingly, age-related auditory loss pertains to poorer cognitive and functional performance compared to people without sensory loss (Rong et al., 2020; Park et al., 2022). Prevalence of dementia increases with increasing severity of hearing loss, while hearing impairment was detected in 94% of people with a cognitive impairment attending a memory clinic (Gold et al., 1996; Huang et al., 2023). In addition, auditory loss is a risk factor for cognitive decline and developing dementia (Bowl and Dawson, 2018; Myrstad et al., 2023; Livingston et al., 2020). Of note, people with dementia and sensory impairment rate their quality of life worse than those without sensory impairment, while burnout and physical exhaustion in care partners can be amplified by communication barriers and greater dependency due to hearing loss in individuals with dementia (Leroi et al., 2019). Age-related hearing impairment in people with cognitive decline poses a significant public health challenge across various healthcare settings. It warrants timely detection and the initiation of necessary interventions for preventing further cognitive decline (Guan et al., 2023; Fu et al., 2022).

The close link between auditory loss and cognitive decline points out that assessing hearing impairment embodies an integral part of the diagnostic workup of cognitive complaints. Indeed, hearing loss screening has been embedded within memory clinic care pathway for individuals at risk of cognitive decline, mainly in tertiary healthcare settings (McDonough et al., 2021; Leroi et al., 2020a), since sensory interventions may potentially improve not only cognition, but also quality of life and neuropsychiatric symptoms of dementia (Lin et al., 2023; Leroi et al., 2019). In addition, hearing impairment may lead to under-performance on cognitive testing, being the backbone of the initial diagnostic procedures at memory clinics, since most of the commonly used assessments rely on auditory cues and questions, although hearing difficulties are rarely taken into account as a confounding factor in such settings (Kim et al., 2023; Lister et al., 2015). Furthermore, people with dementia reported difficulties in understanding the findings of their extensive sensory assessment and the recommendations that followed them (Wolski et al., 2019).

Gold-standard hearing assessment is based on audiometers. However, such equipment is rarely available in primary healthcare settings in high-income countries and frequently not available at all in low- and middle-income countries, which are increasingly confronted with the surge of the numbers of people living with dementia, as two of every three individuals living with dementia reside in low- and middle-income countries (Kalaria et al., 2024). Moreover, the projected increase in global rates of neurocognitive disorders is expected to disproportionately affect such countries. Thus, practical strategies are needed to bypass these challenges to enable the assessment of hearing impairment in people with cognitive decline across the globe, and to safeguard equity in the promotion and protection of auditory and cognitive health.

Valid instruments relying on self-reported auditory difficulties may embody a feasible way to assess the impact of age-related hearing loss on activities of daily living. In people without cognitive impairment, self-reported hearing deficits have a reasonable correlation with objective measurements (Sindhusake et al., 2001). However, people with dementia often may lack insight into the impact of their auditory difficulties (Leroi et al., 2020a). The HHIE is a questionnaire to assess hearing loss in older adults (Ventry and Weinstein, 1982). It comprises 25 items which gauge the perceived effects of hearing impairment on daily life and social settings, as well as the emotional impact of living with hearing loss. The utility of the 25-item Hearing Handicap Inventory for the Elderly (HHIE), as well as that of its 10-item brief version, i.e., Hearing Handicap Inventory for the Elderly-Screening (HHIE-S) (Ventry and Weinstein, 1983; Tomioka et al., 2012), in assessing hearing function in people with dementia have not yet been evaluated. The two instruments can be used interchangeably and their outcomes are congruent with hearing loss level in older individuals (Faraji-Khiavi et al., 2023). A communication partner (spouse) version of the HHIE (HHIE-SP) is available (Leroi et al., 2020b; Regan et al., 2019; Wallhagen et al., 2004). The HHIE-SP comprises 25 items, each of which is analogous to the item of the same number of the HHIE, but addressed to the communication partner. Of note, informant reports of hearing impairment are associated with cognitive decline, too (Vassilaki et al., 2019; Marinelli et al., 2022).

Dementia introduces new challenges to hearing loss assessment through self-reported auditory difficulties, since data collected from individuals with neurocognitive disorders may not accurately reflect their difficulties. Moreover, the 25-item HHIE may constitute an overly lengthy and complicated evaluation process for people with dementia, since cognitive impairment can hinder the individual’s comprehension and attention. Additionally, administering the 25-item tool in primary healthcare, the role of which is pivotal in dementia care (Harris, 2024), may be impractical due to time constraints. To increase accuracy, these data can be complemented with information collected from the care partner through employing the HHIE-SP. In such cases, the tools employed to assess the impact of hearing loss would consist of both the HHIE-SP and the HHIE or the HHIE-S, comprising 50 and 35 items in total, respectively. Nonetheless, such assessments are not compatible with settings beyond tertiary healthcare (e.g., primary healthcare, community private practices). Of note, the different versions of HHIE have not been studied in people with dementia yet.

The aims of the present study are (i) to develop one or more brief instruments based on items of the HHIE and the HHIE-SP, which form a reasonable compromise between comprehensive assessment and brevity and can be administered in different constellations and healthcare settings within the frames of the diagnostic workup of people with dementia, and (ii) to compare their usefulness as well as that of the screening version of HHIE (HHIE-S) in detecting hearing loss severity in people with dementia and hearing loss to HHIE and HHIE-SP.

## Materials and methods

### Participants

The analyses of this study are based on screening- and baseline data of the Sense-Cog Trial, which is a European, multi-center, observer-blind, 36-week long, randomized controlled trial (RCT) of people with dementia with hearing and/or vision impairment and their companions (Sense-cog.eu, 2020; Isrctn.com, 2024; Leroi et al., 2024a). In this RCT, care as usual was compared to a multi-part complex intervention of hearing and vision rehabilitation tailored to each participant dyad, as already described in detail (Leroi et al., 2017; Leroi et al., 2020c; Leroi et al., 2019).

The trial was conducted across eight sites in five European countries: Cyprus (Nicosia), France (Nice), Greece (Athens), Ireland (Dublin) and the United Kingdom (Manchester, Preston and Warrington). Since guidance for inclusion of participants who may lack consent varies across Europe, the principles of the UK’s Mental Capacity Act (2005) were applied to ascertain capacity to consent to the study, using a checklist approach administered to potential study participants by experienced research staff specifically trained in the approach. Care partners who provided consent to participate in the study were asked to represent the wishes of participants lacking capacity and to provide assent, taking on the role of personal consultee.

People with dementia fulfilled ICD-10 criteria for dementia of mild-to-moderate stage, had a score on Montreal Cognitive Assessment (MOCA) (Nasreddine et al., 2005) higher than nine, were 60 years old or older, lived at home and had a care partner (Leroi et al., 2024b). Dementia was defined as an underlying diagnosis of Alzheimer’s disease, vascular dementia, or mixed dementia. The diagnostic workup included structural brain magnetic resonance imaging. People with dementia, who were enrolled in the trial, were on stable cognitive enhancing medication at least 4 weeks prior to screening and had an adult-acquired hearing and/or vision impairment and were willing to accept sensory interventions, if needed. Individuals with unstable, acute, or current psychiatric or physical conditions severe enough to prevent them from participating in the study, complete blindness or severe visual impairment, or deafness (profound hearing loss) were excluded. Those participating in any other trial or having scheduled or urgent treatment or intervention for hearing or vision impairment (i.e., cataract operation already scheduled, treatment for macular degeneration needed) or/and unable to read and write were also precluded.

The inclusion criteria for companions were age 18 or older, serving as an informal caregiver (where providing care is not the person’s primary paid role), such as a significant other of the person with dementia (for example, a family member or close friend), who was either co-resident or in regular contact (on at least a weekly basis), willingness to participate in the study; good command of the language of intervention delivery, as determined by the investigator and being affiliated with a social security system (for France). Companions were excluded if they had an acute or current psychiatric or physical condition severe enough to prevent them from participating in the study, as determined by the investigator and/or were unable to read and write.

### Hearing impairment

Hearing impairment was defined by bilateral hearing difficulties, indicated by failure on a pure-tone hearing screening test in both ears, using the handheld HearCheck— hearing > 35 dB HL over 1- 3 kHz and above in the better ear; the HearCheck screener provided a count of detected signals at or above threshold levels for two frequencies (three levels per frequency) and gave the total number of tones detected from 0 to 6 for each ear. A participant was considered to have a “positive” screen and to be eligible for the trial with any score less than six in both ears. The total number of tones heard in both ears of individuals who heard 10 tones or fewer was considered in the present study.

In addition to the assessment of hearing function by HearCheck during screening, the impact of hearing loss was captured with the HHIE, and subsequently, the HHIE-S, as well as the HHIE-SP at baseline, and these data were taken into account in our analyses. Items of all HHIE versions can be answered with Yes (four points), sometimes (two points), or no (zero points) (Bao et al., 2024). Eight points or less on HHIE-S practically exclude handicap (Purnami et al., 2020).

### Ethics

In Manchester, the study was approved (version 3.0) by the NW Haydock ethics committee on 22 January 2018 and obtained sponsor approval on 8 March 2019. In Nicosia, the study received approval on 27 September 2016 from the Cyprus National Bioethics Committee. In Athens, the Local Ethics Committee of Health Sciences and Scientific Committee of the Eginition Hospital of the National and Kapodistrian University of Athens ethics committee granted a favorable opinion on 24 January 2018. In Dublin, the Saint James Hospital/AMNCH Research Ethics Committee approved the study protocol on 25 October 2018. In Nice, the “Comité de Protection des personnes Sud Est I” gave a favorable opinion on 12 July 2018. Written consent was collected from the participants eligible for the study in line with the national guidance regarding informed consent and clinical research (for individuals with or without capacity to consent) in each of the participating countries. All researchers have been fully trained in Good Clinical Practice (GCP) and mental capacity assessment skills and follow national guidance in their respective countries, such as the Mental Capacity Act (2005) in the UK. If a person lacked capacity, a nominated consultee was asked to deem whether it was in the best interests of the person with dementia to participate.

### Statistical analyses

Exploratory data analysis was performed to provide a comprehensive understanding of the data structure and the characteristics of the sample. More specifically, exploratory data analysis was conducted to examine the descriptive statistics of the analysis, providing insights into the distribution, variability, and relationships among the variables. Eight different proportional odds logistic regression models (Agresti, 2002, 2010) were employed for studying the relationship between the total number of tones heard in both ears (ordinal dependent variable with 11 levels—from 0 to 10) and scores obtained from different versions of the HHIE- and the HHIE-SP instruments, taking into account the demographic characteristics of the person with dementia and his/her communication partner. The different models considered as independent variables the HHIE-S score with and without demographic characteristics, scores from two additional brief versions of HHIE obtained through applying a heuristic procedure for selecting important HHIE- and HHIE-SP items and the demographic factors surviving the heuristic approach described in the following paragraph, the HHIE score with and without demographics, the total scores of both HHIE and HHIE-SP with demographics, and the HHIE-SP score with demographic data. No models with the latter two versions of HHIE without demographic data were computed for the sake of brevity, since their utility would have been lower than that of the models with demographics.

The two additional brief versions were obtained by using stratified repeated random subsampling (stratified bootstrap resampling) (Georgiou et al., 2023; Alexopoulos et al., 2021; Lokhov et al., 2012) to recursive partitioning to training and validation set (90/10 ratio). The procedure was repeated 1,000 times and each time a stepwise approach was used to select the independent variables in the model. The features were arranged based on the percentage of times they were included in the model, i.e., by their importance. Then a forward selection approach was applied by entering one-by-one the most important attributes and assessing the maximum correlation among the included features (Pearsons’s correlations were used for continuous variables, polychoric correlations for polytomous items, tetrachoric correlations for dichotomous items and polyserial or biserial correlations for mixed variables). If the maximum correlation for a newly added variable exceeds a predetermined threshold, the procedure was halted, the specific attribute was excluded from the model and the selection of significant features was concluded. For the present study, two different thresholds were adopted, namely 0.5 and 0.85. Together, these thresholds provide flexibility in balancing feature inclusion and multicollinearity control. The 0.5 threshold allows for moderate correlations permitting some feature overlap while the 0.85 threshold ensures that all the features with some significance, even if they are correlated with other features already included in the model, are included in the model.

Finally, all eight proportional odds logistic regression models were evaluated and compared and ranked by calculating the Mean Squared Error (MSE), one of the most common evaluation measure for regression models (Jacques and Samardžić, 2022; Jayawardena et al., 2023) over 1,000 splits into 90% training and 10% test set, indicating their effectiveness in assessing hearing impairment in individuals with dementia, as reflected in the number of pure tones heard. The results, i.e., the parameter estimates, were then averaged over the splits.

## Results

The study sample encompassed 243 individuals diagnosed with dementia, all of whom had communication partners. 160 participants (65.8%) were diagnosed with dementia due to Alzheimer’s disease, 37 participants (15.2%) with vascular dementia, and 46 participants (18.9%) with mixed dementia (Table 1). The age of the patients ranged from 60 to 93 years, with a mean age of 79.8 years.

**TABLE 1:** Demographic data and clinical characteristics of the study sample.

Individuals with dementia	*N* = 243
Age (years)*	79.8 (5.82) [60–93]
Sex, female, N (%)	127, (52.0%)
**Duration of education**	
Twelve years or less N (%)	164 (67.5%)
Over 12 years N (%)	79 (32.5%)
**Dementia type**	
Alzheimer’s disease N (%)	160 (65.8%)
Vascular dementia N (%)	37 (15.2%)
Mixed dementia N (%)	46 (18.9%)
Use of hearing aid N (%)	73 (30.5%)
**Country of residence N (%)**	
Cyprus N (%)	39 (16.0%)
France N (%)	33 (13.6%)
Greece N (%)	54 (22.2%)
Ireland N (%)	31 (12.8%)
United Kingdom N (%)	86 (35.4%)
Montreal cognitive assessment (MOCA)*	16.7 (3.93) [10–28]
Total number of tones heard*	5.8 (2.46) [0–10]
Tones heard distribution	0 tone: 3 (1.23%)
	1 tone: 5 (2.06%)
	2 tones: 14 (5.76%)
	3 tones: 13 (5.35%)
	4 tones: 58 (23.87%)
	5 tones: 22 (9.05%)
	6 tones: 35 (14.4%)
	7 tones: 20 (8.23%)
	8 tones: 36 (14.81%)
	9 tones: 16 (6.58%)
	10 tones: 21 (8.64%)
Hearing handicap inventory—screening version (HHIE-S)*	9.3 (10.14) [0–40]
Hearing handicap inventory (HHIE)*	18.8 (23.72) [0–98]
**Communication partners**	
Age (years)*	64.7 (13.43) [30–97]
Sex, female N (%)	175 (72.0%)
Sex male N (%)	68 (28.0%)
**Duration of education**	
Less than 10 years N (%)	35 (14.4%)
Ten to 12 years N (%)	73 (30, 0%)
Over 12 years N (%)	135 (55, 6%)
Living with the person with dementia N (%)	146 (60.1%)
Hearing Handicap Inventory communication partner version (HHIE-SP)*	24.5 (23.39) [0–96]

*Mean (standard deviation) [Min-Max].

**FIGURE 1:**
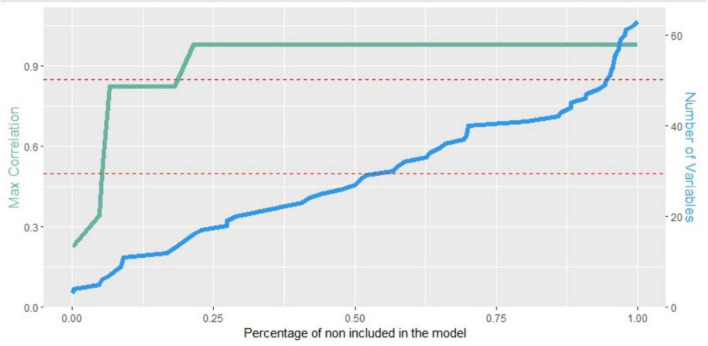
Maximum correlation among the included features (green line) and the number of features included in the model (blue line) with respect to the percentage of times they were not included in the model. The red dashed lines represent the two adopted thresholds.

Two different brief versions of hearing assessment based on items of the HHIE and the HHIE-SP were developed, with the aim to provide a comprehensive assessment while maintaining brevity (Figure 1). The first one, which is ultra-brief, includes only one item of the HHIE (item 7) and one item of the HHIE-SP (item 6) (HHIE-2) (Table 2). The second brief version consists of eight items, i.e., three items from the HHIE (items 7,18, 20) and five items from the HHIE-SP (items 2, 5, 6, 10, 14) (HHIE-8) (Table 2). The models which included as independent variables these two brief versions also considered the age of the person with dementia, being the only demographic factor that was found to be significantly related to the total number of pure-tones heard. All models have been incorporated into the following Google sheet and can be used for estimating the probability of an individual hearing 1–10 pure-tones in the HearCheck screening according to responses to items of different versions of HHIE and demographic characteristics (http://www.des.upatras.gr/amm/economou/HHIE.html). Of note, input data are not stored.

**TABLE 2:** Items of the Hearing Handicap Inventory (HHIE) and the communication partner version of the HHIE (HHIE-SP) included in the 2-item HHIE (HHIE-2) and the 8-item HHIE (HHIE-8).

HHIE	Question
7*	Does a hearing problem cause you to feel “stupid” or “dumb”?
18	Does a hearing problem cause you to want to be by yourself?
20	Do you feel that any difficulty with your hearing limits or hampers your personal or social life?
**HHIE-SP Items**	
2_SP	Does a hearing problem cause him/her to feel embarrassed when meeting new people?
5_SP	Does a hearing problem cause him/her to feel frustrated when talking to members of your family?
6_SP*	Does a hearing problem cause him/her difficulty when attending a party?
10_SP	Does a hearing problem cause him/her difficulty when visiting friends, relatives, or neighbors?
14_SP	Does a hearing problem cause him/her to have arguments with family members?

HHIE, Hearing Handicap Inventory; HHIE-SP, Hearing Handicap Inventory—Communication partner Version; HHIE-2, 2-item Hearing Handicap Inventory; HHIE-8, 8-item Hearing Handicap Inventory—Screening Version.

*Included in both the HHIE-2 and the HHIE-8.

All models computed were ranked based on their MSE values as shown in Table 3. The models considering the HHIE-S and demographic data demonstrated the highest performance (MSE = 6.818), followed by the model including the HHIE-SP alone (MSE = 7.065). The model including HHIE-2 and demographic data proved to be the third most reliable one in our analysis (MSE = 7.254) and was followed by the model comprising HHIE alone (MSE = 7.359), and that considering HHIE-S alone (MSE = 7.586). The performance of the model including the newly developed HHIE-8 exhibited an MSE of 7.740, followed by the comprehensive model incorporating all 50 items of the HHIE and HHIE-SP (MSE = 7.977). The model based on the total score of the HHIE without demographics was the least reliable model (MSE = 9.220) according to the findings of our analyses. Of note, in the model including the HHIE-2 the impact of country of residence was not significant.

**TABLE 3:** Performance of the eight models computed in the study.

Model	Mean squared Error (MSE)	Relative difference from the model including the HHIE-S (%)
HHIE-S and demographics	6.818	−10.1%
HHIE-SP and demographics	7.065	−6.9%
HHIE-2 and age	7.254	−4.4%
HHIE and demographics	7.359	−3.0%
HHIE-S	7.586	−
HHIE-8 and age	7.740	2.0%
HHIE and HHIE-SP and demographics	7.977	5.2%
HHIE	9.220	21.5%

HHIE, Hearing Handicap Inventory; HHIE-S, Hearing Handicap Inventory—Screening Version; HHIE-SP, Hearing Handicap Inventory—Communication partner Version; HHIE-2, 2-item Hearing Handicap Inventory; HHIE-8, 8-item Hearing Handicap Inventory.

## Discussion

Age-related hearing loss is increasingly attracting attention as a crucial factor for maintaining brain health, preventing cognitive decline, social isolation, and loneliness, and easing the burden of care partners of people with neurocognitive disorders (Lad et al., 2022; Sabayan et al., 2023; Leroi et al., 2021). Taking into consideration that audiology services or audiometric examination are not universally available in clinical settings, particularly in low-resource settings such as those in low- and middle-income countries and remote areas of high-income countries, alternative, feasible strategies are urgently needed. Questionnaires assessing the impact of auditory loss may be a pragmatic, alternative strategy.

The analyses of the present study revealed the better accuracy of the HHIE-S and of HHIE-SP when combined with demographic data to capture auditory dysfunction compared to HHIE, HHIE-S and HHIE-SP. HHIE-S combined with demographic data was proved to be helpful in assessing the impact of auditory dysfunction in people with dementia residing in different countries of Europe. People with dementia with higher likelihood to hear fewer than 10 pure-tones based on the model including the HHIE-S and demographic data should be pragmatically referred to audiologists. Those with higher likelihood for hearing 10 pure –tones may be re-assessed with the HHIE-S at follow-up. If a decline in hearing function is detected at follow-up they should be referred to audiometry evaluation. Nonetheless, HHIE-S consists of 10 items and may not be suitable for primary healthcare or community settings, while its length may overwhelm people with advanced stages of cognitive decline. The HHIE-SP, which is based on information derived from the communication partner, proved to be a reliable alternative, ensuring that accurate assessments are still possible through indirect communication. The aforementioned schema of referral to audiometry evaluation or watchful waiting based on the estimation of pure-tones heard by the model including HHIE-SP and demographic data may be followed here, too. However, HHIE-SP is a time-consuming instrument consisting of 25 items. In addition, it can hardly be administered over the phone, if the main care partner does not accompany the person with dementia to his/her visit to the medical facility, where the diagnostic workup of cognitive complaints takes place.

Here, we present an ultra-brief instrument pertaining to pure-tone audiometry screening findings. It relies on one item of the HHIE, one of the HHIE-SP, and the age of the individual with dementia. Even though the model that included HHIE-2 ranked below the HHIE-S and HHIE-SP, it demonstrated sufficient reliability in comparison to the other models (MSE = 7.254, 4.4% relative improvement compared to the HHIE-S), particularly taking into account that the HHIE-S and the HHIE-SP are significantly more extensive and more time-consuming tools compared to HHIE-2. Therefore, HHIE-2 may stand as a more feasible alternative for hearing loss screening or follow-up assessments of the impact of the use of hearing aids in people with dementia in primary and community healthcare settings where time constraints or resource limitations may apply. Furthermore, HHIE-2 addresses the specific challenges of assessing hearing function in individuals with dementia by limiting questions addressed to people with cognitive decline and allowing for care partner input, even over the telephone. While not surpassing HHIE-S or HHIE-SP in terms of accuracy, HHIE-2 can serve as a link between the need for thoroughness and efficiency in low-resource settings in high-income countries but even more in low- and middle- income countries and addresses the importance of equitable and efficient hearing assessments. In addition, country of residence did not exert a significant role in the model including the HHIE-2. In such a way its applicability may not be restricted to European countries where individuals were recruited for the Sense-Cog trial. Unfortunately, the analysis was restricted to people with hearing loss and did not include people without hearing impairment, since they were excluded from the SENSE-cog trial and subsequently they did not complete the HHIE questionnaires. The brevity of HHIE-2 and its examinee- friendliness enables its regular application for follow-up assessments. Examinees with higher likelihood for hearing less than 10 pure-tones according to the model including HHIE-2 and age should be referred to an audiologist and regularly re-assessed with the brief instrument.

The item selection analyses provided evidence for the potential usefulness of a less brief tool consisting of eight items (HHIE-8). Nevertheless, HHIE-8 did not demonstrate sufficient efficacy in assessing hearing impairment in individuals with dementia compared to HHIE-S, HHIE-SP, and HHIE-2. Consequently, it is deemed less suitable for application in clinical settings. Although further research is needed to confirm our findings in larger independent samples, this quite unexpected deviation in the performance between HHIE-8 and HHIE-2, considering the more thorough capturing of auditory function through the eight-item tool compared to HHIE-2, could hypothetically be attributed to noise caused by inconsistencies in the reliability of responses of individuals with dementia and their communication partners as well as to overfitting of the models due to the relatively small sample size (Numbers et al., 2022; Huang and Yang, 2023). This is in line with the low reliability of the model including all 50 items of both the HHIE and the HHIE-SP, suggesting that a more extensive assessment does not necessarily improve performance in regard to people with dementia and their care partners.

Models including demographic data consistently demonstrated higher performance compared to those restricted to questionnaires. Particularly age was found to be inversely related to the number of tones heard by the individuals with dementia and hearing loss. This finding seems to reflect the age-related nature of hearing dysfunction in our study sample. In addition, as expected the use of hearing aids was significantly related to less pure tones heard. The impact of age on the evaluation of hearing loss in clinical practice was previously described (Choi et al., 2019). In the models in which a significant relationship between the number of tones heard and the age of the communication partner was found, the relationship was positive. This positive correlation may be a spurious finding. Nonetheless, it warrants further investigation, since it may shed light on the complex interplay between hearing loss and social withdrawal or even isolation of the person with hearing loss and the age of the primary communication partner (Motala et al., 2024). In the case of our study, this interplay is further perplexed by the effects of dementia on communication (Folder et al., 2023).

The recruitment at multiple sites, the availability of pure-tone screen data derived by the accurate HearCheck screener (Fortnum et al., 2016), and the investigation of the utility of questionnaires assessing hearing function for the first time in people with dementia are the advantages of our study compared to previous efforts in this field. Based on their association with the findings of pure-tone audiometry screening, brief or more extensive questionnaires when combined with demographic data are more accurate in assessing hearing function than HHIE, HHIE-S, and HHIE-SP according to the findings of the present study. Of note, since the study sample consisted of people with mild-to-moderate dementia, it is very likely that the detected association between data collected through HHIE items and audiometry findings may not be applicable in people with severe dementia, taking into account the severe cognitive and functional impairment of these people, which impedes their ability to understand and report on the impact of their hearing difficulties.

The limitations of the study include the recruitment of participants at specialized centers, the relatively small sample size, the inclusion exclusively of individuals with hearing loss and not also of people with dementia and no hearing loss, which did not enable the ascertainment of the accuracy of the tools in identifying the hearing function status for instance through calculating sensitivity and specificity, the consideration of hearing screen data in the analyses and not of the findings of a detailed audiometric assessment and the lack of biomarker-based diagnoses (Frisoni et al., 2024) or histopathological verification of the underlying cause of dementia. Taking into account the fact that clinical diagnoses of dementia type are not always confirmed at autopsy and biomarkers of Alzheimer’s disease are abnormal in many cases even in non-Alzheimer dementia types (Alexopoulos et al., 2018; Skillbäck et al., 2015), while detecting the cause of the dementia syndrome in community and primary healthcare settings is less of an issue than timely diagnosis of dementia (Bradford et al., 2009; Perna et al., 2019), we intended to create models that are universally applicable regardless of the dementia subtype, in order to ensure broad utility and practicality. Another shortcoming of the study may be the consideration of individuals with dementia coming from only five European countries, which may limit the applicability of the instruments to only these countries. However, it is noteworthy that country of residence did not exert a significant impact on the model including HHIE-2. Moreover, HHIE is a measure of the social and emotional impacts of hearing loss, which are assumed to be as a proxy of hearing problems and audiometrically ascertained hearing loss, and not of hearing function itself. Nevertheless, self-report measures of the impact of hearing loss are commonly used to identify hearing loss, because they are correlated with audiometric findings (Choi et al., 2019). In addition, the questionnaires that were used for assessing the impact of hearing loss are based on the judgment capacity and critical thinking of the examinees. Interestingly, even very subtle age-related changes in cognitive function seem to negatively affect judgment (Boyle et al., 2012). Thus, it cannot be discounted that study participants may have misjudged the extent of the social and emotional impacts of hearing loss, while their estimation may have been misguided by neuropsychiatric symptoms such as depressive symptoms or apathy which are very common in dementia (Siafarikas et al., 2017). Of note, it was recently shown in a prospective cohort study that self-reported hearing loss was not associated with increased dementia risk, even though up to 32% of 8-year incident dementia could be attributable to audiometric hearing loss (Ishak et al., 2025). Hence, people with incipient dementia may not be fully aware of their own hearing difficulties. Finally yet importantly, since pure- tone audiometry is a psycho-acoustic test method, its reliability and accuracy may be negatively influenced by factors, such as reduced alertness and impaired cognitive function, which are parts of the clinical phenotype of dementia (Hoff et al., 2023). Thus, future studies employing central auditory processing assessment may provide additional, valuable insights.

To sum up, two conclusions emerge from our study. Firstly, (i) questionnaires assessing the impact of hearing loss correlate with findings of pure-tone audiometry screening in people with dementia and age-related hearing loss and (ii) the 10-item HHIE-S combined with demographic data, the communication partner 25-item HHIE-SP again with demographic data, and the ultra-brief HHIE-2 combined with the age of the person with dementia form pragmatic tools to assess auditory function in variable European settings. HHIE-2 may be suitable for applicability in different countries, since in a European cohort it is not affected by the country of residence of the individual with dementia. Nonetheless, these encouraging results need replication and validation before further conclusions can be drawn. Future research should focus on further validating such tools in diverse populations, including people with dementia and no hearing loss, and exploring their integration into routine clinical practice.

## Data Availability

The raw data supporting the conclusions of this article will be made available by the authors, without undue reservation.
